# Nanostructured Titanium Nitride and Its Composites as High-Performance Supercapacitor Electrode Material

**DOI:** 10.3390/nano13010105

**Published:** 2022-12-25

**Authors:** Nazish Parveen, Mohammad Omaish Ansari, Sajid Ali Ansari, Pramod Kumar

**Affiliations:** 1 Department of Chemistry, College of Science, King Faisal University, P.O. Box 380, Hofuf, Al-Ahsa 31982, Saudi Arabia; 2 Center of Nanotechnology, King Abdulaziz University, Jeddah 21589, Saudi Arabia; 3 Department of Physics, College of Science, King Faisal University, P.O. Box 400, Hofuf, Al-Ahsa 31982, Saudi Arabia; 4 Functional Materials Laboratory, Department of Chemistry Prof Rajendra Singh (Rajju Bhaiya) Institute of Physical Sciences for Study and Research, V. B. S. Purvanchal University, Jaunpur 222003, Uttar Pradesh, India

**Keywords:** titanium nitrides, electrode material, energy storage, nanostructured, synthesis, nanocomposite

## Abstract

Electrochemical supercapacitors as an energy storage device have become trademark in current electronic, medical and industrial applications, as they are sources of impressive power output. Supercapacitors supply fast power output, suitable to cover the energy demand of future electronic devices. Electrode material design is a subject of intense research in the area of energy development and advancement, due to its essential role in the electrochemical process of charge storage and the cost of capacitors. The nano-dimensions allow for more electroactive sites, different pore size distributions, and a large specific surface area, making nanostructured electrode materials more promising. Electrode materials based on metal oxides, metal nitrides, and metal carbides are considered ideal for highly efficient electrochemical supercapacitors. Recently, much effort has been devoted to metal nitride-based electrodes and their diverse compositions as they possess higher electrical conductivity and better corrosion resistance, electrochemical stability, and chemical reactivity. Among these, titanium nitride (TiN), possesses high electrochemical stability, outstanding electrical conductivity, and a unique electronic structure. Nanocomposites based on titanium nitrides are known to deliver higher electrochemical performance than pristine nanostructured TiNs due to potential synergetic effects from both the materials. In this paper, recent advancements made in the field of nanostructural TiN electrode materials for SCs are reviewed along with their challenges and future opportunities. Additionally, some of the major techniques involved in the synthesis process are discussed, along with some basic concepts.

## 1. Introduction

Uncontrolled use of fossil fuels not only leads to their rapid depletion but also raises substantial concerns about global warming and energy shortages. The progress made possible by the use of such energy sources is not said to be long-term and sustainable [1,2,3]. Henceforth an urgent need has arisen for the clean, green and economic generation and utilization of energy. Renewable energy sources are the preferred solution to such concerns. Electric energy production through solar, wind and hydro energy is said to be the future of energy [4,5]. However, the non-continuous supply of such energy sources is a major limitation. Therefore, electric energy storage is equally important as its generation [6]. Among several energy storage systems, electrochemical energy storage (EES) is the most popular and efficient method for storing renewable energy, such as solar and wind energy [7,8]. Batteries and supercapacitors (SCs) are the two most popular EES technologies, both of which are known for their low cost and high performance [9]. Batteries can deliver high energy densities in the range of 10–265 Wh kg^−1^ and are used in a variety of applications including portable devices, electric vehicles, and powering homes. However, because of their short life cycle and low power density, they are unsuitable for a variety of applications that require a large amount of energy in a short period [10,11]. A supercapacitor (SC) is a well-renowned technology equipped with the novel features of high-power density (>10 kW kg^−1^), elongated cycle life (>10^5^), rapid charge–discharge processes and low cost. The device is promising in a wide range of applications ranging from hybrid electric vehicles, space crafts, memory back-up devices and portable electronic devices [12,13]. The charge storage mechanism of SCs relies on two principles, known as electrostatic storage of charges and the faradaic process of charge storage. The resulting capacitances from electrostatic charge storage are known as electric double-layer capacitance, in which charges are stored at the interface of electrode and electrolyte through physical attractive forces between the opposite polarities. This is a very fast charge storage process and the SCs possessing such capacitances are known to deliver the highest power densities. Electric double-layer capacitors (EDLCs) consist of electrodes primarily made of carbonaceous materials, which hold the key to high conductivity, large specific surface area, and long lifespan [14,15]. On the other hand, pseudocapacitance arises from the faradaic charge storage process in which the actual transfer of charges takes place at the interface of electrode and electrolyte. The capacitors which own such a charge storage mechanism are called pseudocapacitors (PCs) and possess higher energy densities than EDLCs [16]. Conducting polymers and metal oxides are the most preferred choice of electrode materials for PCs [17,18]. However, EDLCs have poor energy density delivery, whereas PCs have lower power densities. To combine the benefits of both the EDLCs and PCs into a single capacitor, a third category of hybrid capacitors came into existence which are known to possess both high energy and power densities [19]. Electrode materials are considered a prominent component, one that plays a major role in the EC performance of a SC device. Carbonaceous materials and pseudocapacitive materials (metal oxides and conducting polymers) are the two most common forms of electrode materials for SCs [20,21]. The low cost, good electrochemical stability, and large surface area of carbon-based electrode materials are well-known novel features. The availability of carbonaceous materials in a variety of forms has led to the commercialization of EDLCs with high power capabilities. Activated carbon, carbon nanotubes, graphene, and carbon aerogel are used as electrode materials in EDLCs [22]. These are enriched by various dimensional structures, porosity, large surface areas, and conductivity. Liu et al. for example, investigated a wide range of carbon-based electrode materials for SCs [23]. However, in addition to their distinct characteristics, these materials have poor specific capacitance capability and a low energy density. The pseudocapacitive electrode materials are known for good specific capacitance storage capability, high redox activity, and high energy density. Several metal oxides that have been explored for SCs are ruthenium oxides, iron oxides, manganese oxides, zinc oxides, nickel oxides, and cobalt oxides [24,25,26]. Although the presence of a large number of oxidation states enriches their working performance, the inferior electrochemical stability on continuous charge–discharge cycles and poor power performance are the major drawbacks. Conducting polymers are also considered pseudocapacitive materials due to their high specific capacitance capability, low cost, eco-friendly nature, and high capacitive nature. These include polyaniline, polypyrrole, polythiophenes, polyvinyl alcohol, and polyindole [27,28]. However, on continuous intercalation and de-intercalation of electrolyte ions to their surface, a significant change in the volume is observed, which results in inferior electrochemical stability. In this domain, transition metal nitrides (TMNs) have attracted much research attention as SC electrode materials [29,30,31]. The TMNs possess metallic structures, with their interstitial sites occupied by nitrogen atoms. The bonding between metals and nitrogen greatly affects the physical and chemical nature of the complete structure. Such configuration provides an enriched electrical conductivity comparable to metals, higher redox activity than their metal oxide counterparts, and excellent electrochemical stability. Several groups have explored the potential application of TMNs in energy storage [32,33,34]. In this series, the explored TMNs are vanadium nitrides, molybdenum nitrides, titanium nitrides, and niobium nitrides [35]. Although all of these metal nitrides possess unique behavior for energy storage, Titanium nitride has been a pressing topic of research for SC electrodes [36,37]. This is promising due to the presence of several characteristic features, such as high electrical conductivity comparable to that of metals (4 × 10^3^–5 × 10 ^4^ S cm^−1^), good electrochemical stability, extreme mechanical strength, and low production cost [38]. Interestingly, it has been shown that the electrochemical performance of TiN is boosted with the introduction of oxygen vacancies at its surface due to the growth of TiOx by air exposure. This signifies that the charge storing behavior of TiN greatly depends upon its surface chemistry [39]. The surface-dependent properties of TiN open a new window through which to explore its electrochemical performance by varying different parameters such as surface area, porosity, morphology, the density of materials, and crystal structures. The charge storing characteristics of TiN involves the combined effect of electrostatic charge storage as well as the reversible faradaic charge-storage process [40]. Along with the choice of suitable electrode materials such as TiN, the SC device performance greatly depends on the configuration and design of the electrodes. In recent years, it has been observed that the nanostructuring of electrodes provides a large surface area, porosity, abundance of redox-active sites, conductivity, and electrochemical stability.

**Figure 1:**
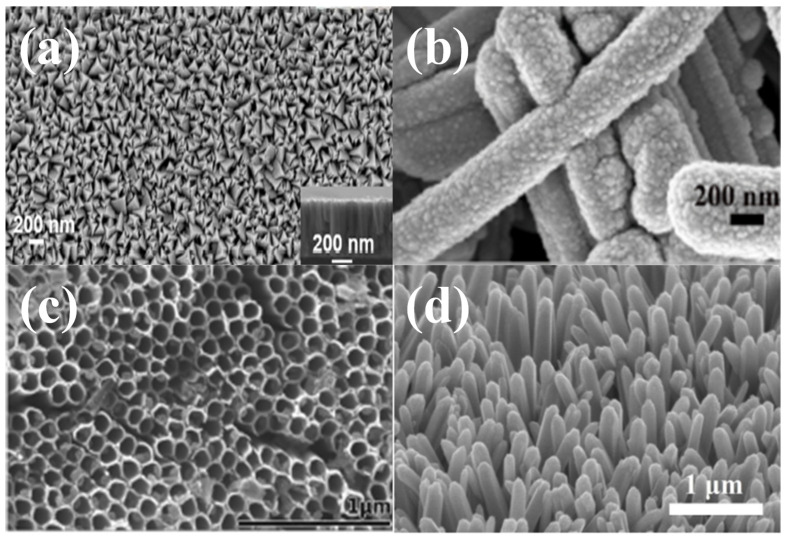
(**a**) FE-SEM image of nanopyramid shaped TiN thin film synthesized by sputtering process [45], reproduced with permission, copyright 2018, Elsevier. (**b**) Magnified SEM image of rod-like Si_60_ NWs–TiN electrode [46], reproduced with permission, copyright 2021, Elsevier. (**c**) Mesoporous SEM images of the top surface view of TiN nanotube arrays [47], reproduced with permission, copyright 2014, Royal Society of Chemistry. (**d**) SEM image of the TiN nanowire arrays on the carbon nanotube fibers [48], reproduced with permission, copyright 2018, American Chemical Society.

Nanostructuring of electrode materials provides short ionic diffusion pathways and helps in increasing the specific capacitance [41,42,43]. Therefore, the electrode should be designed in such a way that electrolyte ions can quickly travel through the conducting channels. The different techniques are used to realize the 0D, 1D, 2D, 3D, and hierarchical nanostructures for electrode materials [44]. TiN has been synthesized in a range of unique nanostructures, as shown in Figure 1 [45,46,47,48]. The above-mentioned TiN nanomorphologies are rich in electroactive sites, and their stable nano geometries provide short paths for ions to move rapidly, efficiently, and reversibly to the surface of active materials. Although sufficient work has been carried out on the synthesis of different nanostructures of TiN, no review has summarized the potential application of nanostructured titanium nitride for SCs. In this critical and comprehensive review, the progress achieved in the fabrication of nanostructured TiN-based SCs is explored in depth. Additionally, the fabrication of electrode materials requires a synthesis approach and therefore the understanding of each synthesis technique is of equal importance. Herein, we have provided a brief introduction of different synthesis techniques used in the fabrication of SC electrodes with suitable examples. Following that, the application of various forms of TiN, such as pure TiN, TiN–carbonaceous composites, TiN–metal oxides, and TiN–polymers composites, is reviewed along with their major challenges, to provide an in-depth overview of the prospective use of nanostructured TiNs for SCs.

## 2. Synthesis Techniques for Nanostructured Titanium Nitride

The development of innovative and stable potential electrode materials relies on the synthesis techniques used. Different synthesis techniques are employed in the fabrication of nanostructured TiNs for SC electrodes, a few of which are briefly explained here.

### 2.1. Hydrothermal–Solvothermal Technique

**Figure 2:**
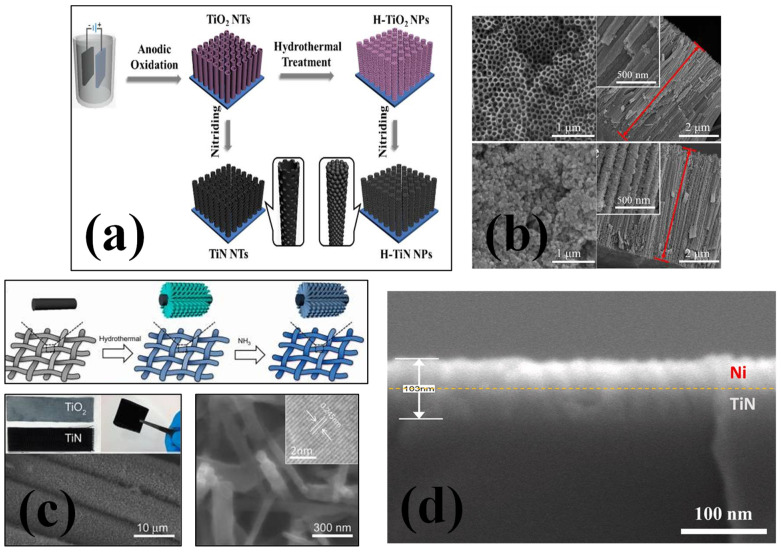
(**a**) The synthesis mechanism and (**b**) SEM images from different surfaces for TiN nanotubes and H-TiN nanopillars [52], reproduced with permission, copyright 2013, Royal Society of Chemistry. (**c**) Schematic diagram illustrating the synthesis process for TiN nanowires grown on carbon cloth substrate and magnified SEM and TEM images of TiN nanowires [54], reproduced with permission, copyright 2012, American Chemical Society. (**d**) The SEM cross sectional image of TiN–Ni nanocomposite [64], reproduced with permission, copyright 2021, Elsevier.

One of the most often used methods for fabricating nanostructured SC electrodes is the solution-based hydrothermal synthesis method, which is simple and environmentally benign [49]. This method allows for the development of a wide variety of materials with nano morphologies, ranging from zero to three-dimensional architectures. The closed system approach, which uses high temperatures and pressures, produces materials with excellent purity, homogeneity, crystallinity, and controlled morphology [50]. The synthesized materials’ physical and chemical natures are dependent on their solvent properties, with the supercritical phase allowing particle formation due to the fast reaction kinetics. When water is replaced by another solvent, the same process becomes solvothermal [51]. Although pristine TiN offers excellent electrical conductivity and rapid charge–discharge rates, the poor cyclability and inferior capacitance are the major drawbacks [32]. To resolve such problems, Qin et al. employed a facile hydrothermal method to synthesize nanopillars of hierarchical TiN nanoparticles. The binder-free electrode is fabricated via the nitration of TiO_2_ nanotubes and results in H-TiN nanopillars with a surface area of 23.1 m^2^g^−1^ and diameter under the range of 100–150 nm, with a length of 6 µm, as shown in Figure 2a,b. As an electrode, the H-TiN NPs showed a capacitance of 69 Fcm^−3^ at a current density of 0.83 Acm^−3^. Both the large specific surface area and increased electroactive sites are attributed to the remarkable EC performance [52]. To provide extra stability to TiN nanostructures, coating with other potential materials is highly encouraged. A hydrothermal technique effectively provides the formation of such a configuration. For example, Lu et al. emphasized that the continuous intercalation–deinteracalation of electrolyte ions during the cycling process results in the structural degradation of TiN electrodes. The group used hydrothermal technique to grow a thin layer of amorphous carbon onto the TiN nanowires grown onto the carbon cloth. The resulting TiN@C electrode delivered an improved specific capacitance of 124.5 Fg^−1^ at a current density of 5 Ag^−1^, improving on the 107 Fg^−1^ for a pure TiN electrode. Additionally, for a long cyclic process (15,000 charge-discharge cycles), the TiN@C electrode retained 91.7% of its initial capacitance [53]. Liu et al. explored the mechanism behind the capacitance loss of hydrothermally grown TiN nanowires on carbon cloth. An XPS data analysis revealed that the irreversible oxidation of TiN during the continuous electrochemical cycling process is the reason the capacitance fades. Interestingly, the use of poly vinyl alcohol (PVA–KOH) as a polymer electrolyte provided a high stability to the TiN solid-state SC over 15,000 cycles with a volumetric energy density of 0.05 mWh cm^−3^. The morphology and the synthesis process of TiN nanowires is shown in Figure 2c [54]. Sun and co-workers reported a fiber SC based on a core–shell TiN@C nanotube-shaped electrode. After performing a complete nitrification of the TiN core, a hydrothermal process was used to coat a shell of carbon on the core of the TiN. Such a hybrid configuration resulted in a capacitance of 2.4 mF cm^−1^ at a 10 mV s^−1^ of scan rate. Additionally, high capacitance retention of 80% was achieved after running 10,000 charge–discharge cycles. The carbon coating is attributed to the enhanced performance of PVA–KOH gel electrolyte-based flexible SC [55]. The electrochemical performance of TiN nanostructures can also be enhanced by combining the TiN with other metal nitrides. For example, vanadium nitride is promising for energy storage due to its high theoretical capacitance value and good conductivity. Therefore, its incorporation with TiN can further boost the electrochemical performance. Recently, Wei et al. reported a bimetallic nitride TiVN for SC application synthesized through solvothermal process. The mesoporous hollow spheres morphology of the TiVN composite contributes to the improved electronic conductivity and specific capacitance with a value of 729 Fg^−1^ at 2 Ag^−1^ of current density. The synergetic contribution from both the counterparts is attributed to the remarkable performance [56]. The above reviewed literature signifies the potential use of the hydrothermal technique in the fabrication of nanostructured TiN-based SC electrodes. Although the cyclic stability over a large number of cycles is greatly improved by following different strategies, the limited specific capacitance is still a challenge.

### 2.2. Magnetron Sputtering Technique

Magnetron sputtering has evolved to become an advanced technique for the deposition of highly uniform and pure thin films of different metal oxides, nitrides and sulfides [57]. The technique is utilized in several application areas such as memory devices, optical devices, semiconducting devices, decoratives and electrical devices. Magnetron sputtering consists of RF and DC sputtering with the difference in the working mechanism. In a normal sputtering process, a target whose film is to be deposited on the substrate is bombarded by highly accelerated and energetic ions. This results in the emission of atoms from the target which get deposited as thin films onto the surface of the substrate placed at a height, just opposite to the target. A high control over the operating conditions, such as pressure (10^−3^ mbar–10^−2^ mbar), temperature and voltage, results in the formation of defect-free thin films [58,59,60]. The synthesis of TiN nanostructures using other techniques such as the hydrothermal process and thermal decomposition involves the participation of other gases such as oxygen, ammonia, and the requirement of extremely high temperatures (~800 °C). Depositing TiN nanostructures directly onto the substrates helps in improving the electrochemical performance due to the absence of any binder and conducting agents [61]. Magnetron sputtering facilitates the fabrication of highly conformal and pure binder-free thin films of TiN directly onto the substrates. For example, Arif et al. used the DC sputtering at 80 W of power supply to obtain the pyramid-shaped thin films of nanostructured TiN with an atomic percentage ratio of Ti:N as 54.24:15.26%. A symmetric SC device based on TiN thin films showed a capacitance of 112 Fg^−1^ at 1 Ag^−1^ with awesome capacitance retention of 92.6% for a long number of cycles of 30,000 with a remarkable energy density of 26.28 Wh kg^−1^ and power density of 12.9 kW kg^−1^. The nanopyramid configuration is attributed to the enhanced performance [45]. Recently, the demand for micro supercapacitors has risen due to their reliability and their ability to power other on-chip devices. However, good cyclic stability and improved energy density are the major requirements for enhanced performance. In this direction, the deposition of TiN thin films through magnetron sputtering adds value to the fabrication of micro SCs. For example, Achour et al. used a sputtering-based micro-fabrication process for the fabrication of TiN thin films onto flat silicon substrates. The films with controlled porosity delivered a volumetric specific capacitance of 146.4 F cm^−3^ with negligible decay of capacitance over 20,000 cycles. The performance is attributed to the doping of nitrogen into TiO_2_ layers, porosity and thickness of the deposited film [38]. In another study, a micro-SC based on binary metal nitride composite TiVN thin films was fabricated by using DC magnetron sputtering technique. A relation between specific capacitance and Ti–V atomic ratio has been explored and it was found that at an optimized 1:1 Ti–V ratio, a maximum areal capacitance of 15 mF cm^−2^ is delivered in the presence of 1M aqueous KOH electrolyte. Moreover, the TiVN thin film-based electrode showed negligible capacitance decay over 10,000 cycles. A synergetic contribution from both the counterparts attribute to such good capacitance retention [62]. The concept of flexible and wearable electronic devices has attracted much research attention in recent years [63]. In this sequence, thin films with nanostructures and porosities provide short ionic diffusion pathways and facilitate the motion of electrolyte ions. Recently, Sial et al. fabricated a flexible solid-state SC device based on TiN–Ni thin film deposited by DC magnetron sputtering at a power of 150 W under 10 m Torr of pressure for 30 min. The porous TiN thin films grown possess a thickness of 103 nm with high uniformity, shown in Figure 2d. The presence of a thin layer of Ni enhanced the conductivity and electrochemical stability of the TiN–Ni electrode, with the assembled device showing a specific capacitance of 10.21 mF g^−1^ at 5 mV s^−1^ of scan rate with good energy and power densities [64].

### 2.3. Electrochemical Deposition

**Figure 3:**
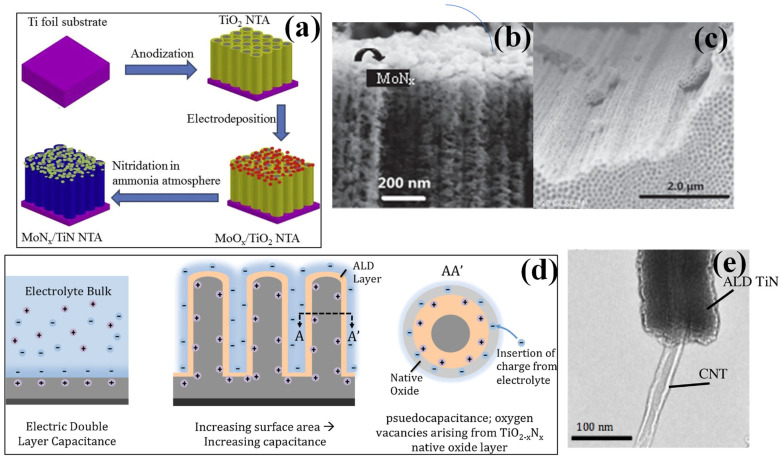
(**a**) The experimental procedure and synthesis mechanism of MoNx−TiN NTAs using electrochemical deposition process, (**b**) SEM image of side view for TiN nanotubes, and (**c**) SEM images of the side view of MoNx−TiN nanotube arrays respectively [72], reproduced with permission, copyright 2016, Elsevier. (**d**) Conceptual representation showing the effect of increased surface area on the capacitance and a rise in pseudo capacitive effect. (**e**) TEM image of ALD-grown TiN coated with the carbon nanotube showing a uniform coating with a layer of around 20 nm respectively [79], reproduced with permission, copyright 2016, Elsevier.

This deposition technique is well known for rapid synthesis, low cost, non-toxic by-products, and high purity for the synthesis of a variety of materials varying from metal oxides to conducting polymers. The electrodeposition process is carried out using several techniques, such as cyclic voltammetry, double-pulse deposition and potential step, with the help of reference, counter and working electrodes [65,66,67]. It results in the formation of a variety of nanostructures from 0D to 3D with porosity varying from micropores to mesopores. The technique also holds potential for depositing nanostructured TiN thin films on different substrates which are used as binder-free electrodes for SCs [68]. It has been demonstrated that the cyclic performance of TiN-based thin films improves with the growth of an oxide layer on its surface. Therefore, it is interesting to explore the relation between capacitance and surface activation by the formation of an oxide layer onto the surface of TiN thin films. For example, Gray et al. tried to understand the effect of surface oxide layers on the EC performance of TiN thin films. Following the electrodeposition process, the titanium foils were converted into TiN thin films by the anodization process in ammonia. Their CV analysis predicted that, with the increase in oxidative treatment, the initial cycles would not show an increase in capacitance values. However, the subsequent cycles showed greater capacitance with 39 Fcm^−2^ at a high scan rate of 100 mV s^−1^ [69]. The EC performance is highly influenced by the morphology of electrodes. For instance, 1D nanostructural morphology provides an efficient and rapid transport of ions through the conducting channels [70]. A study based on the fabrication of co-axial 1D MnO_2_–TiN nanotube arrays through the facile electrodeposition process has been reported by Dang et al. The TiN nanotubes were synthesized using anodization of Ti foil using ammonia. The 1D MnO_2_–TiN nanocomposite showed an improved specific capacitance of 681 Fg^−1^ at a high current density of 2 Ag^−1^ with only 3% capacitance decay after 1000 continuous cycles. The high conductivity of TiN nanotubes and redox active behavior of MnO_2_ contributed significantly to the EC performance [71]. The EC performance of TiN nanostructures can be further enhanced by merging the TiN with other metal nitrides. For instance, molybdenum nitride has been explored as a potential active electrode material due to its layered structure (γ-Mo_2_N), high electric conductivity (0.2 S cm^−1^), enriched electrochemical stability and high charge storing ability. Xie et al. tried to incorporate MoNx with TiN as MoNx–TiN NTA with the help of electrodeposition process followed by nitration process in ammonia. The average diameter of the nanotubes was found to be 110–130 nm with a length of around 4 µm (Figure 3a–c). The synergetic contribution of both MoNx and TiN contributed to the improved capacitance of 121.50 mF cm^−2^ with a maximum rate capability of 93.8% over 1000 continuous charge–discharge cycles [72]. TiN–Li_4_Ti_5_O_12_ NTAs were reported by the group of Xie et al. Applying the lithiation process of TiO_2_, the Li_4_Ti_5_O_12_ NTAs were synthesized with a further coating of TiO_2_ sol and annealing in an ammonia atmosphere. The TiN-Li_4_T_5_O_12_ delivered an enhanced specific capacitance of 143.83 Fg^−1^ at a current density of 0.5 Ag^−1^ with good capacitance retention of 82.41% over 1000 cycles. A solid-state SC showed a capacitance of 40.45 Fg^−1^ at 0.5 Ag^−1^ of current density [73]. The above reviewed literature signifies the potential use of electrochemical deposition for fabrication of SC electrodes.

### 2.4. Atomic Layer Deposition Technique

**Figure 4:**
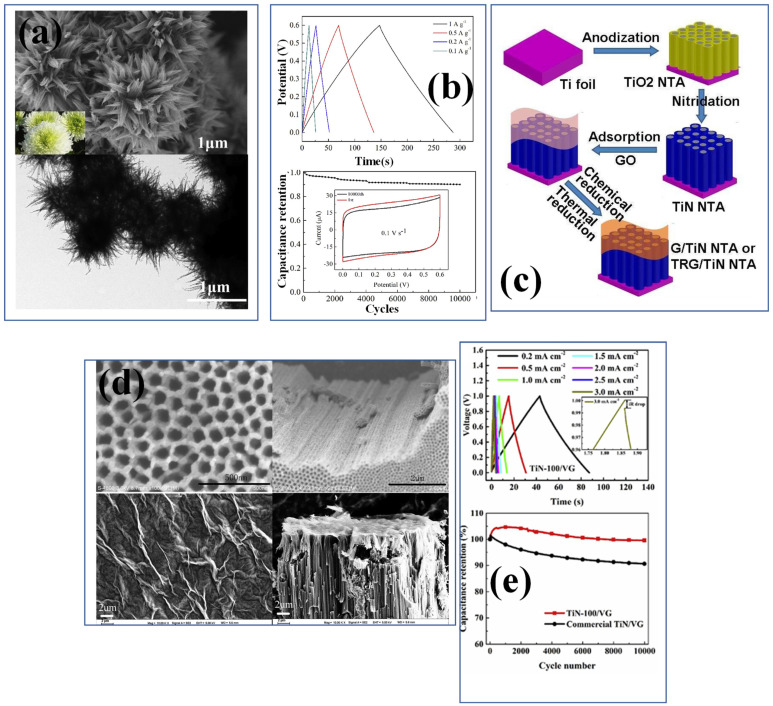
(**a**) The SEM–TEM image of chrysanthemum-like CL−TiN and GCD, (**b**) capacitance retention curve of a CL–TiN nanostructure [89], reproduced with permission, copyright 2018, Elsevier. (**c**) The synthesis mechanism of G–TiN nanotube arrays and (**d**) SEM images of top surface and cross-section view of TiN nanotubes and graphene coated TiN nanostructures [97], reproduced with permission, copyright 2014, Royal Society of Chemistry. (**e**) The GCD and capacitance retention curve for TiN-100 vertically grown graphene and commercial TiN-VG [98], reproduced with permission, copyright 2019, Elsevier.

This vapor deposition technique is well known for developing thin films of various nanostructures, development of large surface area current collectors, hard resistive coatings and fabrication of semiconducting and electrical microchip devices [74,75]. The process can be applied to a variety of materials including carbonaceous materials, metal oxides, metal sulfides and metal nitrides. This technique offers a precise control over the porosity, thickness, and purity of the thin film grown onto different substrates, which helps in improving the electrochemical performance of the device [76]. The one step conformal surface coatings provided by the ALD also allows the fabrication of composite electrodes. Therefore, to improve the cyclic performance, electrical conductivity, electrochemical stability, energy and power densities of SCs, the ALD technique for the fabrication of thin-film-based electrode materials is highly encouraged [77]. The major limitations for other deposition techniques in the synthesis of TiN nanostructures are the difficulty in achieving a precise control over the geometry, high temperature requirement and long processing time. The ALD process also supports the synthesis of TiN–carbon composite for SC electrodes. A large surface area with porosity is the foremost requirement for the high performance of SCs. Carbon nanotubes (CNTs) are considered to have the most potential as an EDLC’s electrode material [78]. Their use is promising in the development of SCs possessing high voltage, high energy and power density. Composites based on the incorporation of a tubular backbone such as TiN, with the outer surface coated by a highly conducting pseudocapacitive material, leads to the formation of a new generation of SCs. The three main factors that can illustrate this are: (1) the good settlement and bonding of metal nitride counterparts with carbonaceous materials, (2) the large surface area with porous network that provides a smooth conducting channel for electrolyte ions to diffuse rapidly, and (3) the way that the composite gains extra strength when less volumetric changes occur in the material on continuous charge–discharge cycles. A good example of fabricating a TiN–CNT nanocomposite through highly conformal ALD technique has been reported by Kao et al. On performing a 400-cycle run of ALD, a thick TiN layer of around 20 nm is well grown on each CNT. This results in a 500% increment of the EC capacitance of an ALD-grown TiN-CNT-based device—81 mF cm^−2^ as opposed to 14 mF cm^−2^—over CNT-based electrodes (shown in Figure 3d,e). The use of CNTs increases the porosity and surface area while TiN thin film grown over CNTs enhances the electronic conductivity and electrochemical stability of the composite [79]. On-chip SCs have recently gained a lot of attention from both the research and the industry points of view. To power portable electronic devices with printed boards a smooth and controlled flow of electricity is highly important. However, it becomes quite difficult to assemble the powering devices at microscale level. The ALD technique has been found to be quite important in the fabrication of on-chip micro-SC devices. For example, Grigoras et al. successfully grew an ultrathin TiN layer by utilizing an ALD technique. The nanostructured TiN thin film (thickness 15 nm), grown onto porous silicon substrate (thickness 1.5 µm) improved the specific capacitance due to an increase in the surface-to-volume ratio (Figure 4). The assembled on-chip SC delivered a remarkable specific capacitance of 15 F cm^−3^, with negligible loss in capacitance after 23,000 continuous charge–discharge cycles [80].

### 2.5. Molten Salt Technique

The molten salt technique is a co-effective and very simple method for the synthesis of the various ceramic materials and oxide powders. In this synthesis procedure molten salt enhances the fluidity of the reaction components in the liquid phase, which is responsible for improving the rate of the diffusion and stopping the aggregation of the particles during the synthesis procedure. This results in a shortened reaction time and lower temperatures required for synthesis. Recently, molten salt techniques have been used for the synthesis of various materials [81,82,83,84].

Metal nitrides such as vanadium nitrides, titanium nitrides, iron nitrides etc. have been synthesized through a molten salt-based synthesis method. In the past, Ding et al. have reported TiN whiskers can be synthesized through the molten salt media on a graphitic substrate. In this synthesis method, titanium metal (Ti), NaF and NaCl are utilized as raw materials. The authors used molar ratios of the Ti and graphite of 1:1, 1:2, 1:3 and 1:4, whereas the NaCl and NaF weight ratio was 10:1. Firstly NaF and NaCl were added equally to the Ti and graphite and were then mixed in an alumina crucible used to place the powdered mixture and maintain the temperature at 1100 to 1400 °C for 3 hours in an argon atmosphere [85]^.^ The formatted TiN whisker with a diameter of 500–600 nm also modified the surface area of the graphite. Recently Guan et al. have reported porous three-dimensional high surface area metal nitrides such as VN, MoN, WN and TiN using the mild molten salt route. They used enviro-friendly ZnCl2 as the molten salt phase and maintained a temperature of 290 C at 1 atm [86].

## 3. Different Forms of Nanostructured TiNs for Supercapacitor Electrodes

### 3.1. Pure Nanostructured TiN for SC Electrodes

Nanostructured titanium nitride is well known for its electronic conductivity comparable to that of metals (3.70 × 10^6^ S m^−1^), cubic crystal structure, high melting point of 3050 °C and density ~5.40 g cm^−3^ [87]. The material is reported to synthesize in a variety of nanostructural morphologies with the help of different techniques. To enhance the energy density of TiN-based electrodes several approaches are applied. For example, the concept of a binder-free electrode is helpful in the rapid transport of electrolyte ions through the conducting networks. Fabrication of TiN as a binder-free electrode avoids the use of conducting agents and binders and consequently enhances the EC performance [88]. This strategy can be used for the development of thin and foldable metallic foils as flexible electrodes for SCs and are of keen importance in wearable electronic devices. Hou et al. reported a flexible solid-state SC based on chrysanthemum-like TiN as an electrode synthesized by the facile hydrothermal method. The unique morphology of CL-TiN possesses a diameter of 2 µm with a nanorod width of 200 nm and provides an efficient ion diffusion channel (Figure 4a,b). The assembled SC device delivered a volumetric capacitance of 7.48 F cm^−3^ with an energy density of 0.34 mWh cm^−3^ at a current density of 0.05 A cm^−3^. Moreover, remarkable capacitance retention of 136% was achieved after running 20,000 charge–discharge cycles. The 3D flower-like morphology, high conductivity and good electrochemical stability are attributed as reasons for the remarkable EC performance [89]. In another study, Achour et al. provided evidence of the influence of nitrogen doping on the surface of TiN thin films with charge storing ability. The grown films using DC reactive magnetron sputtering resulted in pyramid-shaped nanostructures with a film thickness of 760 nm. An increase in β-N dopant yielded a threefold increase in areal capacitance of 8.2 mF cm^−2^ with negligible capacitance loss after 10,000 cycles [90]. The large surface area of the electrode materials is quite prominent for high performance SC electrodes since accumulation of more charges at the electrode surface result in higher capacitance value. Additionally, if TiN can be synthesized with a large specific surface area, then it becomes quite useful. Following a similar approach, Choi et al. successfully synthesized nanocrystalline TiN with a large surface area of 128 m^2^g^−1^ using a two-step halide approach. The different nanostructures were obtained by varying the heat treatment temperatures and were evaluated for their EC performances [91]. Although nanostructured TiN is explored as a promising SC electrode due to its high electric conductivity and mechanical strength, the limited specific surface area and lesser number of electroactive sites for charge storage are some limitations that need to be resolved.

### 3.2. TiN–Carbon Based Nanocomposite for SC Electrodes

High power density and long cycle life are the key properties of SCs while the inferior energy density is the major limitation that needs to be overcome. To overcome this, the concept of nanocompositing an electrode material has been highly recommended [53,55]. A composite based on TiN–C holds excellent EC properties with a large surface area, various porosities and high conductivity. A long cyclability test of 10,000 cycles for TiN–rGO has been performed by Haldorai et al. following a two-step process; the nanoparticles of TiN were uniformly dispersed into an rGO matrix and delivered a capacitance of 415 Fg^−1^ at 0.5 Ag^−1^ of current density. Good interaction and dispersion of the TiN nanoparticles with rGO sheets were attributed as reasons for the enhanced EC performance [92]. Wang et al. utilized a green chemical synthesis route to fabricate a TiN–C composite-based SC electrode. Incorporating carbon with TiN, the surface area was increased to 148 m^2^g^−1^ with the composite delivering a specific capacitance of 159 Fg^−1^ at a low current density of 0.5 Ag^−1^. The EC performance was attributed to the well dispersed TiN nanoparticles at an annealing temperature of 700 °C in the carbon matrix, which had a large electroactive surface area [93]. The inferior electrochemical stability and poor cyclability are the major problems associated with metal nitrides such as TiNs. The application of large surface area carbon materials with high cyclic performance and stability have been proven to improve the EC performance. Among various carbonaceous materials, graphene is the most researched electrode material with the highest potential due to its versatile properties such as high electrical conductivity, large specific surface area and good mechanical strength [94,95]. Incorporating graphene with TiN can boost the EC performance due to the synergetic contribution from both the materials. Considering a similar concept, Lee et al. successfully reported a nanocomposite of TiN–graphene with a large portion of Ti in the composite. Synthesized through the two different routes, the TiN–G composite delivered an improved capacitance over the TiN–G-TE nanocomposite. The presence of a large number of oxygen vacancies in the TiN–G composite was attributed to the higher capacitance it had over TiN–G-TE [96]. Synthesis routes can affect the EC performance of an electrode and a good example of this concept is given by Tian et al. For their study, TiN nanotube arrays grown onto nickel foam and bare nickel foam were considered as substrate to coat graphene onto the surfaces (shown in Figure 4c,d) which were then referred to as G–TiN NTAs and G–NF. The difference in reducing GO into graphene through chemical reduction and thermal reduction resulted in a major difference in the capacitance—333.7 Fg^−1^ for G–TiN and 198.7 Fg^−1^ for G–NF. A solid-state symmetric SC device based on G–NF NTA showed an energy density of 34.2 Wh kg^−1^ at a higher power density of 11.3 KW kg^−1^. The high electronic conductivity of TiN and the large surface area of graphene were attributed as reasons for the enhanced EC performance [97]. An interconnected porous structure with high electrochemical stability and purity is the most demanding characteristic property of high performing nanostructures of TiN-based electrodes. The thermal and chemical synthesis routes are most utilized but the use of templates, hazardous chemicals and extreme high temperature conditions over long periods are the major drawbacks of these synthesis routes. In contrast, the plasma-related synthesis methods under high vacuum conditions are quite effective due to their short reaction times, impurity free environments and uniform depositions. The application of such growth techniques have been emphasized by the work reported by Qi et al. Utilizing the transferred arc method, the TiN nanoparticles with cubic crystal structure and a size of 5–20 nm were successfully grown onto vertical graphene to fabricate a TiN–VG hybrid electrode. With the high voltage window of 1.8 V, the composite delivered a remarkable cyclic stability of 89.5% over 10,000 continuous charge–discharge cycles (Figure 4e). The good crystallinity, high metallic content, and porous morphology were attributed as reasons for the good cyclic performance [98]. Although the TiN–C composite holds promising application for SC electrodes, reports based on this material are scarcely reported. Thus, the material needs to be further explored.

### 3.3. TiN–Conducting Polymer-based Composite for SC Electrodes

**Figure 5:**
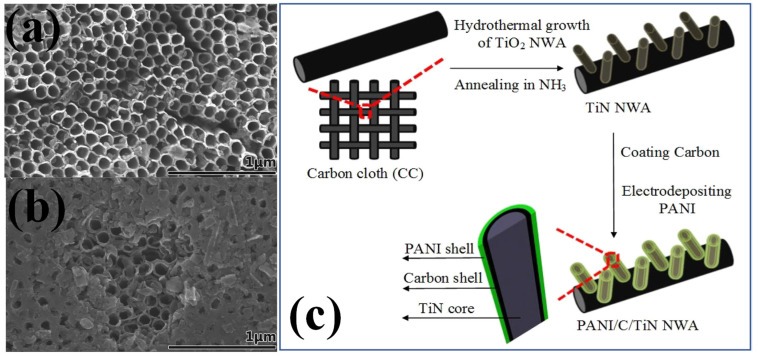
(**a**) The SEM images of the top view of TiN nanotubes and (**b**) PPy coated TiN nanotubes [47], reproduced with permission, copyright 2014, Royal Society of Chemistry. (**c**) The synthesis mechanism of PANI–C–TiN NWA grown onto CC substrate [70], reproduced with permission, copyright 2015, Elsevier.

Recently the organic–inorganic composite for SC electrodes have been widely explored [99,100]. The organic part consists of conducting polymers (CPs), showing the charge storage from pseudocapacitance while the inorganic part consists of metal oxides, metal sulfides or metal nitrides [101]. CPs are promising due to their high energy storage capability, environmentally benign nature, large operating potential window, low cost and ease of fabrication. The different polymers that hold potential uses in SCs are polypyrrole, polypropylene, polyvinyl alcohol, polyacryl amide etc. [102]. These are suitable for organic–inorganic composite form due to their easy doping mechanism and excellent electric conductivity. Among various metal nitrides, TiN is highly suited for such combination because of its excellent electronic conductivity, high mechanical strength, and good electrochemical stability. Such a composite includes the noble features of both the types of materials and therefore help in increasing the overall performance of the device. Electrochemical deposition is the preferred choice of fabrication technique for a CPs–TiNs composite-based electrode [103]. In this sequence, conducting polypyrrole (PPy) is the most promising CP due to the high electrical conductivity, effective interface area, porous nature and nanoscale dimension architecture. These can be synthesized in a range of nanostructures such as nanofibers, thin films, nanosheets etc. [104]. The incorporation of nanostructured TiNs in the composite also acts as a substrate material with a large surface area, high conductivity and good electrochemical stability. They facilitate a smooth and rapid diffusion of electrolyte ions to the surface of active electrode materials and serve as a backbone for the composite [98]. Following the same approach, Du et al. successfully synthesized a PPy–TiN nanocomposite using the electrodeposition approach and compared the EC performance with a PPy–TiO_2_ composite. The TiO_2_ nanotube arrays were converted into TiN nanotubes at an annealing temperature of 800 °C in the presence of NH_3_. The resulting TiN nanotubes possessed a diameter of 100–110 nm with wall-coated PPy thin layers on the surface (shown in Figure 5a). The PPy–TiN composite delivered a highly specific capacitance of 1265 Fg^−1^ compared to 382 Fg^−1^ for PPy–TiO_2_ at a small current density of 0.6 Ag^−1^. This represents the high conducting nature of TiN NTAs and the same is ascribed for the increase in the EC performance [47]. Incorporation of a p-type polymer such as PPy and an n-type polymer such as PANI with a highly conducting TMN such as TiN greatly helps in improving the charge-storing ability of SCs. This configuration provides effective and short pathways for the rapid diffusion of electrolyte ions. For instance, Xie et al. fabricated a solid-state flexible SC by incorporating PPy–TiN–PANI nanotube array as a composite electrode. The presence of two different types of polymers induced more electroactive sites for the storage of electric charges with the TiN nanotube arrays providing a good electric conducting channel. The PPy–TiN–PANI composite synthesized through the pulse voltammetry and cyclic voltammetry outperformed with a capacitance of 1471.9 Fg^−1^ in presence of 1M H_2_SO_4_ electrolyte at a current density of 0.5 Ag^−1^. Even at a high current density of 10 Ag^−1^, the hybrid material delivered a high capacitance of 1077.4 Fg^−1^. All the counterparts of the hybrid composite contributed significantly to the remarkable EC performance [105]. Low mass transfer resistance and high conductivity of the electrode is key to obtaining good efficiency in EC energy storage. In this perspective, the concept of self-standing current collectors with directly grown active materials on their surfaces has gained considerable attention. This provides fast electronic and ionic transport, large specific surface area, good wettability of electrode surface by electrolyte ions and high electrochemical stability. The overall synergetic contribution leads to high energy and power density of the SC device. A unique combination based on phosphomolybdic acid–polyaniline–titanium nitride core–shell as composite electrode has been successfully fabricated by Lu et al. (shown in Figure 5b). The good dispersion of both the polymers with each other and the high conducting backbone of TiN in the form of binder-free electrodes fabricated by an electrodeposition process resulted in a specific capacitance delivery of 469 Fg^−1^ at a current density of 1 Ag^−1^ with the device showing an energy density of 216 Wh kg^−1^ at an operating voltage window of 1.5 V [106]. A ternary nanocomposite based on PANI–MnO_2_–TiN as electrode material synthesized by a combination of hydrothermal and electrochemical deposition is reported by Xia et al. A three-layered structure as TiN NWA of diameter 10–30 nm uniformly covered with a layer of MnO_2_ and then a PANI layer result in an enhanced capacitance of 674 Fg^−1^ at a 1 Ag^−1^ of current density. The introduction of MnO_2_ increased the pseudocapacitance effect and was attributed as reason for the increase in capacitive performance [107]. The application of CPs provides flexibility to the SC electrodes and is quite useful in wearable electronics devices. Among various CPs, polyaniline (PANI) is the most versatile due to its low cost, high specific energy, good theoretical capacitance and environmentally benign nature. However, the major problem associated with such polymers is the degradation in their structure due to swelling and shrinking after a long cycling process [28]. This limits their application for the long-term use of flexible SCs. Several research directions have been suggested to overcome this limitation and one of these is to incorporate them with other carbon, metal oxide or metal nitride species. Such an incorporation could provide mechanical strength to the CPs. Along with this, a novel nanoarchitecture such as a core–shell structure is of great importance. For instance, Xie et al. developed a ternary nanocomposite PANI–C–TiN, incorporating the combine properties of a polymer, a carbonaceous material and titanium nitride. The multicomponent material is synthesized in a shell–shell–core-shaped nanostructure with TiN NWA of diameter 40–60 nm. The fabricated novel electrode delivered a remarkable specific capacitance of 1092 Fg^−1^ at a current density of 1 Ag^−1^ with high capacitance retention of 98% over the 2000 continuous cycles. The presence of a middle carbon layer is attributed as reason for the extra strength provided to PANI along with the positive contribution from all individual components of the hybrid composite [70]. Peng et al. reported a hybrid co-axial PANI–TiN–PANI NTA as SC electrode through electrochemical deposition of PANI on the outer, upper and inner surface of TiN NTAs. The electrode showed a specific capacitance of 242 mF cm^−2^ with a good capacitance retention of 83% over 3000 charge–discharge cycles. The 3D nanoporous structure with large surface area was responsible for the improved electrochemical performance [108]. Nanostructured TiN supported CPs have been found to be promising electrode material for SCs. However, there is still a large challenge in synthesizing such hybrid nanostructures with various dimensions, porosities and morphologies. The fabrication techniques are only limited to electrochemical deposition. Thus, to further explore the potential use of TiN–CPs hybrids for SCs, other low-cost and scalable synthesis methods should be researched and utilized. Polymers in SCs are not only applied in composite form with TiN but also work as templates in the synthesis. TiN synthesized through this process possesses higher capacitance then by other methods. This is due to the fact that template-grown TiN possesses a large specific surface area, various pore size distributions and conducting channels with short pathways. For example, Kin et al. synthesized mesoporous TiN thin films by using poly(vinyl chloride)-graft-poly(oxyethylene methacrylate) (PVC-g-POEM) polymers as a template. The flexible SC device that was fabricated did not lose the capacitance even on bending and delivered a capacitance of 266.8 Fg^−1^ with high cycling stability [109].

### 3.4. TiN–Other Materials-Based Composite for SC Electrodes

Due to the excellent electrical conductivity and electrochemical stability, nanostructured TiNs provide a conducting framework to hold various transition metal oxides (TMOs) such as MoO_x_, MnO_2_, NiCo_2_O_4_ etc. [36,42]. The presence of TiN offers mechanical strength and rapid transfer of electrolyte ions which helps to enhance the capacitance nature of the hybrid electrode material. Another unique property of TiNs are their easy tunability with other metal oxides, resulting in extra stable nanostructures. The metal oxides have been widely explored as pseudocapacitive electrode materials. They possess higher energy density and capacitance than EDLC electrodes (>2000 Fg^−1^) but suffer from poor electronic conductivity and low specific surface area. One of the strategies to improve the electric conductivity of metal oxides is to incorporate them with conducting TiNs as a conducting substrate or as a composite material [46]. Peng et al. synthesized a double-layer coated MoO_x_ NTAs over highly conducting TiN cores to assemble MoO_x_–TiN–MoO_x_ as a composite electrode, one that comprises the high conductivity and pseudocapacitance of a TiN core with the pseudocapacitance arising from a TiN shell, as well as the electroactive sites. This in turn improved the capacitance and cycling performance, with the specific capacitance delivery of 97 mF cm^−2^ at a scan rate of 1 mA cm^−2^. The electrochemical stability of MoOx contributed significantly to the symmetrical SC device, showing a volumetric capacitance of 24 Fcm^−3^ and the device exhibited the full capacitance retention over 10,000 cycles. The dual-layered MoO_x_ NTA over the thin TiN provided high mass loading with a large surface area and was attributed as the reason for the increase in the device’s overall performance [110]. Fibers possessing mesoporous nanostructural materials are the most suitable for SCs, in which flexible electrodes with desired porosities are an innovative research avenue for the development of high-performance SC electrodes. This configuration provides a large surface-to-volume ratio with more electroactive regions and short diffusion ionic pathways. Among several metal nitrides, Vanadium nitride is also promising due to its improved capacitance storage. However, it possesses good electronic conductivity [111]. Incorporating the two different metal nitrides is a good way to enhance the rapid transportation of electrons and ions with enlarged charge storing ability. In this perspective, a composite based on core–shell TiN–VN fibers as flexible SC electrodes has been explored by Zhou et al. Using the co-axial electrospinning method, highly porous and large surface area core–shell fibers with an outer diameter of 600 nm were synthesized. Both the materials contributed significantly and delivered a specific capacitance of 247.5 Fg^−1^ at a scan rate of 2 mV s^−1^ due to their fibrous structure that provided a large area of access to the electrolyte ions. The electrode maintained a capacitance retention of 88% over 500 cycles. The improved specific capacitance delivery and good rate capability were ascribed to the presence of TiN and VN in the composite [112]. The poor energy density of SCs is the major limitation that hampers their wide-scale application. The device energy density is proportional to the voltage window and capacitance. Therefore, optimization of these two parameters through the use of suitable electrode materials and device configuration is the forefront need. The fabrication of an asymmetric SC device is the most emphasized strategy to obtain a large energy density. Along with this, the choice of electrode materials such as nanostructured TiN is the perfect combination. Recently, Wei et al. fabricated an asymmetric SC device based on a bimetallic nitride TiNbN as cathode and VN as anode with 0.5 M H_2_SO_4_ as electrolyte. The magnetron sputtered grown bimetallic nitride TiNbN as binder-free electrode and delivered a capacitance of 59.3 mF cm^−2^ at a scan rate of 1 mA cm^−2^ with outstanding cyclability after 20,000 cycles. The asymmetric configuration led to an extended voltage window of 1.6 V with enhanced energy and power densities as 74.9 mWh cm^−3^ and 8.8 W cm^−3^ respectively. The synergetic contribution from Ti and Nb were ascribed as reason for the boost in EC performance [35]. Although the TiN–other materials-based electrodes discussed above have excellent electrochemical characteristics, the majority of the nanostructured TiN reported work as a current collector or a scaffold to load other materials. This leads to an unnecessarily high electrode weight, as well as the inability to properly utilize nanostructured TiNs, resulting in inferior capacitive performance.

### 3.5. TiN-based Electrode Material Used in Flexible–Wearable SC

Flexible energy storage devices are fundamental to the development of next generation wearable, compact and portable electronics for medical, military and civil applications such as health tracking devices, computers, television, or flexible displays on phones. Flexible supercapacitor devices are highly attractive in comparison with batteries as they combine the inherent high power density (≥10 kW–kg), fast charging–discharging capability, longer life cycles and good mechanical flexibility. In conventional supercapacitors they consist of the outer case, the current collectors in the form of the metal foils, and cathode and anode electrodes in the electrolyte separated by the ion transport layer. In the case of flexible supercapacitors, the highly conducting and flexible carbon network serves as both the electrodes and current collector. Therefore, the architecture of the flexible supercapacitors is highly conducting and lightweight and is further simplified for portable electronic devices. Thus, self-supported and flexible electrodes have been paid intensive attention. Deposition of the active material on the flexible textile is one of the most effective and widely reported methods to fabricate flexible free-standing electrodes. Recently, highly conductive transition metal nitrides such as TiN, VN, MoN, FeN have been used in flexible SCs due to their high capacitance (100–1340 Fg^−1^). Among these, TiNs have attracted more attention due to their higher conductivity, which is closer to pure metal. For example, Lu et al. have reported free-standing TiN nanowires grown on a carbon cloth using a two-step synthesis method. Firstly, they uniformly grew 100 to 200 nm TiO_2_ nanowires on carbon cloth via a seed-assisted hydrothermal synthesis method. After that, these nanowires were thermally annealed in a NH_3_ atmosphere and with a constant temperature in the range of 700–1000 °C to obtain the TiN. The fabricated flexible free-standing TiN electrode shows an excellent electrochemical performance. In this work the authors claim that the flexible solid-state TiN SC retained 83% retention after 15,000 cycles. The electrochemical performance of the flexible solid-state TiN SC could open up new opportunities for a flexible solid-state SC based on TiN and its composites [55]. Recently, Bin et al. fabricated porous tin nitride paper as an effective electrode for ultrafast charging–discharging SC. The fabricated TiN paper achieved excellent conductivity. In this work the authors fabricated a porous and highly conductive TiN paper as a flexible electrode which enabled the rapid electron transport and ion diffusion that are required for ultrafast charging [113]. Table 1. summarizes the synthesis methodology, obtained morphology and the electrochemical parameters of few metal nitrides.

## 4. Conclusions

To increase the performance of supercapacitors (SCs) enough to power advanced electronics and other technologies, the development of innovative and promising electrodes is the most pressing need. Recent research carried out in the development of SCs has emphasized the nanostructuring of electrode materials. These changes are vital due to the following facts: (1) a smaller particle size with sub-nanoscale dimensions provides a large specific surface area for the generation of electrons and the accumulation of ions on the electrode surface; (2) nano-dimensional architecture facilitates short paths for the diffusion of electrolyte ions, resulting in higher wettability of electrode surface; (3) nanostructuring of electrode materials strengthens the whole electrode, which helps in providing electrochemical stability; (4) fabrication of SC electrodes with a wide range of pore-size distributions with various dimensions are only possible by nanostructuring, and is therefore very important for accessing the maximum surface area of electrodes by electrolytes; and (5) nanostructuring provides high surface-to-volume ratio to electrodes, which increases their capacitance. Along with the strategy of nanostructuring electrodes, choice and optimization of an electrode material is equally important. Among several types of electrode materials, transition metal nitrides are of huge importance due to their high conductivity, structural stability and remarkable electrochemical performance. Titanium nitride (TiN) has recently been explored for potential use in SC electrodes. The material is well known for its excellent electronic conductivity, comparable to that of metals, and its good mechanical strength. The nanostructures of TiNs add value to their novel characteristics in terms of higher specific energy, specific power, cyclic stability and large operating potential window. Considering the promising features of nanostructural TiNs, it is important to summarize the latest advancements made in nanostructural TiNs, with the help of different synthesis techniques, incorporating their importance, advantages and complications. In this review, a comprehensive overview of SC performance based on nanostructural TiNs was provided, including a brief introduction of some techniques involved in the fabrication of electrodes. The EC performance of pristine nanostructured TiNs can be enhanced by incorporating these with other materials such as carbonaceous materials, metal oxides, metal sulfides and conducting polymers. The composite hybrid form of nanostructural TiNs incorporates the synergetic effect of all the counterparts, resulting in extraordinary conductivity, electrochemical stability and mechanical flexibility. The concept of free-standing and binder-free nanostructural TiNs or composites avoids the usage of any binder or conducting agent and provides ultra-long cycling performance to the electrodes. However, in comparison with other SC electrode materials, TiN has received little attention, and, as a result, research on this material is limited. The lack of understanding of the original and fundamental mechanism of charge store behavior in the crystal lattice is the cause of this scarcity. The process of their capacitance decay is similarly poorly understood. As a result, there are numerous research gaps in existing nanostructured TiNs that need be investigated in order to increase the EC performance of SCs. Apart from the obvious advantages of TiNs, there are also some intrinsic drawbacks limiting their practical applications. There is a challenge in large-scale controllable synthesis. Fabrication of TiNs with controlled/specific structures and crystal planes usually requires multistep processes and high temperature treatment, which make the process costly, complicated, or even uneconomical.

## Figures and Tables

**Table 1:** Synthesis methodology, morphology and electrochemical parameters of a few metal nitrides.

	Electrode Material	Synthesis Method	Morphology	Specific Capacitance	Energy Density	Ref
1	MnO_2_–TiN	Anodization/ammonia/electrodeposition	Nanotube arrays	681 Fg^−1^ at 2 Ag^−1^	-	[73]
2	Two-dimensional titanium carbide ‘clay’	Hydrothermal/ammonia reduction	Nanowires	124.5 Fg^−1^ at 5 Ag^−1^	-	[114]
3	Mesoporous vanadium nitride	Sample annealing thermal-assisted anodizing method	Nanoporous film	291.7 mF cm^−2^	56.3 mWh cm^−2^ energy density	[115]
4	Mesocrystal vanadium nitride nanosheets	Ammonia reduction	Nanosheets	1937 mF cm^−3^	--	[116]
5	Vanadium nitride–carbon nanotube nanocomposites	Sol–gel/ammonia reduction	Nanoparticles	270 Fg^−1^	--	[117]
6	NixCo_2_x(OH)_6_x–TiN Nanotube Arrays	Anodization/ammonia/electrodeposition	Nanotube arrays	2543 Fg^−1^ at 5 mV s^−1^	--	[118]
7	TiN@GNS	ALD	Vertically aligned nanosheet −3 energy density		0.51 mWh cm^−3^ energy density	[119]
8	Titanium nitride	Sol–gel	Nanoparticles	407 Fg^−1^ at current density 1 Ag^−1^	211.4 mW cm^−3^ power density	[41]
9	Holey tungsten oxynitride nanowires	Hydrothermal/ammonia reduction	Nanowires		1.27 mWh cm^−3^ energy density	[120]

## Data Availability

Not applicable.
